# Identifying Symptoms Prior to Pancreatic Ductal Adenocarcinoma Diagnosis in Real-World Care Settings: Natural Language Processing Approach

**DOI:** 10.2196/51240

**Published:** 2024-01-15

**Authors:** Fagen Xie, Jenny Chang, Tiffany Luong, Bechien Wu, Eva Lustigova, Eva Shrader, Wansu Chen

**Affiliations:** 1 Department of Research and Evaluation Kaiser Permanente Southern California Pasadena, CA United States; 2 Pancreatic Cancer Action Network Manhattan Beach, CA United States

**Keywords:** cancer, pancreatic ductal adenocarcinoma, symptom, clinical note, electronic health record, natural language processing, computerized algorithm, pancreatic cancer, cancer death, abdominal pain, pain, validation, detection, pancreas

## Abstract

**Background:**

Pancreatic cancer is the third leading cause of cancer deaths in the United States. Pancreatic ductal adenocarcinoma (PDAC) is the most common form of pancreatic cancer, accounting for up to 90% of all cases. Patient-reported symptoms are often the triggers of cancer diagnosis and therefore, understanding the PDAC-associated symptoms and the timing of symptom onset could facilitate early detection of PDAC.

**Objective:**

This paper aims to develop a natural language processing (NLP) algorithm to capture symptoms associated with PDAC from clinical notes within a large integrated health care system.

**Methods:**

We used unstructured data within 2 years prior to PDAC diagnosis between 2010 and 2019 and among matched patients without PDAC to identify 17 PDAC-related symptoms. Related terms and phrases were first compiled from publicly available resources and then recursively reviewed and enriched with input from clinicians and chart review. A computerized NLP algorithm was iteratively developed and fine-trained via multiple rounds of chart review followed by adjudication. Finally, the developed algorithm was applied to the validation data set to assess performance and to the study implementation notes.

**Results:**

A total of 408,147 and 709,789 notes were retrieved from 2611 patients with PDAC and 10,085 matched patients without PDAC, respectively. In descending order, the symptom distribution of the study implementation notes ranged from 4.98% for abdominal or epigastric pain to 0.05% for upper extremity deep vein thrombosis in the PDAC group, and from 1.75% for back pain to 0.01% for pale stool in the non-PDAC group. Validation of the NLP algorithm against adjudicated chart review results of 1000 notes showed that precision ranged from 98.9% (jaundice) to 84% (upper extremity deep vein thrombosis), recall ranged from 98.1% (weight loss) to 82.8% (epigastric bloating), and *F*_1_-scores ranged from 0.97 (jaundice) to 0.86 (depression).

**Conclusions:**

The developed and validated NLP algorithm could be used for the early detection of PDAC.

## Introduction

Pancreatic cancer is the third leading cause of cancer deaths in the United States, with 50,550 estimated deaths in 2023 [1]. Pancreatic ductal adenocarcinoma (PDAC), which accounts for 90% of pancreatic cancer cases, is the most common form of pancreatic cancer. The age- and sex-adjusted incidence has continued to increase, reaching 13.3 per 100,000 in 2015-2019, and the overall 5-year survival remains poor at only 12.5% [2]. Despite technological advances, diagnosis of pancreatic cancer remains very late, with more than 50% of patients having distant metastases at the time of diagnosis [2-4].

Patient-reported symptoms are often the trigger for evaluation that eventually leads to a diagnosis of pancreatic cancer [5,6]. The reported prevalence of symptoms associated with PDAC has largely varied due to many factors, such as study design and data sources [6-10]. Additionally, previously published studies have been based on patient surveys [6,7] or structured electronic health records (EHRs) [8-10]. However, structured data can be inaccurate [11,12] and incomplete [13], especially for signs and symptoms. On the other hand, signs and symptoms are frequently collected and documented in the clinical notes by care providers via free text within the EHRs. Therefore, extracting signs and symptoms from clinical notes offers a key opportunity for the early detection of pancreatic cancer, which can lead to more timely interventions that improve survival.

Identification of PDAC-related symptoms from clinical notes based on EHRs is a challenge because signs or symptoms are typically not well-documented in a structured format within an EHR system, and specific techniques are required for data processing and analysis. Natural language processing (NLP), a field of computer-based methods aimed at standardizing and analyzing free text, processes unstructured data through information extraction from natural language and semantic representation learning for information retrieval, classifications, and predictions [14]. Numerous innovative NLP applications have been developed across various clinical domains in support of medical research, public health surveillance, clinical decision making, and outcome predictions [15-19]. Early NLP applications have largely focused on rule-based approaches [15,16], while recent NLP applications utilize state-of-the-art machine learning [17] or deep learning approaches via transformer learning models [18-20]. Rule-based NLP techniques have been widely used to extract signs and symptoms from free-text narratives in past years [21-26]. To the best of our knowledge, we are not aware of previous studies systematically analyzing pancreatic cancer–related symptoms from clinical notes via NLP. The purpose of this study is to develop and validate a comprehensive NLP algorithm and process to effectively identify PDAC-related symptoms prior to diagnosis within a large integrated health system.

## Methods

### Study Setting

Kaiser Permanente Southern California (KPSC) is an integrated health care system providing comprehensive medical services to over 4.8 million members across 15 large medical centers and more than 250 medical offices throughout the Southern California region. The demographic characteristics of KPSC members are diverse and largely representative of the residents in Southern California [27]. Members obtain their health insurance through group plans, individual plans, and Medicare and Medicaid programs and represent >260 ethnicities and >150 spoken languages. KPSC’s extensive EHR data contains individual-level structured data (ie, diagnosis codes, procedure codes, medications, immunization records, laboratory results, and pregnancy episodes and outcomes) and unstructured data (ie, free-text clinical notes, radiology reports, pathology reports, imaging, and videos). KPSC’s EHR covers all medical visits across all health care settings (eg, outpatient, inpatient, and emergency department). Clinical care of KPSC members provided by external contracted providers is captured in the EHR through reimbursement claim requests. 

### Ethical Considerations

The study protocol was reviewed and approved by the KPSC Institutional Review Board (approval no. 12849) with a waiver of the requirement for informed consent.

### Study Population Identification

**Figure 1:**
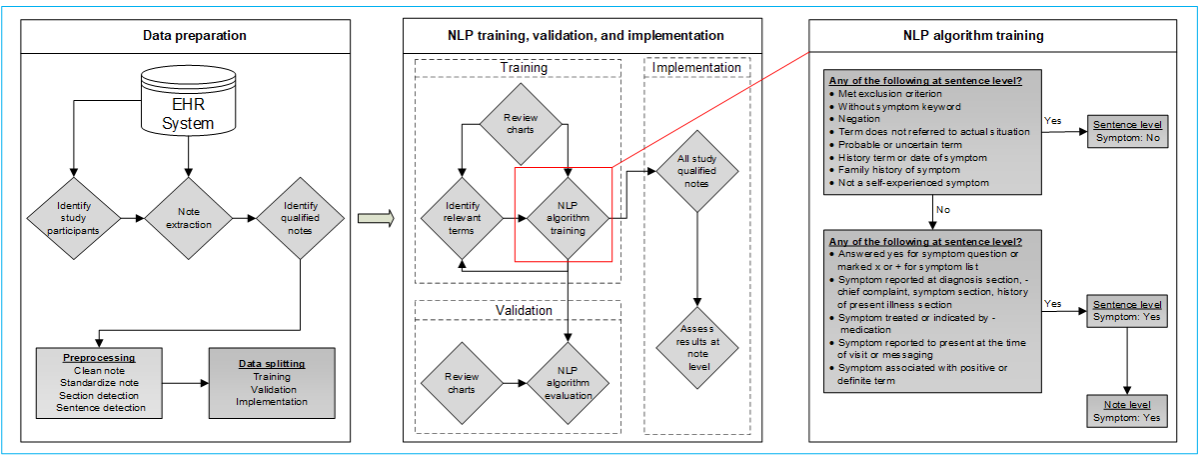
Schematic diagram of the NLP algorithm to identify the pancreatic ductal adenocarcinoma–related symptoms. EHR: electronic health record; NLP: natural language processing.

This study was a nested case-control study of KPSC patients aged 18-84 years between 2010 and 2019. Patients diagnosed with PDAC were identified through KPSC’s cancer registry. Patients with a history of acute or chronic pancreatitis, without a clinic-based visit within 3 to 24 months prior to the diagnosis, with chemotherapy or infusion treatment, or with less than 20 months of health plan enrollment or pregnancy within 2 years prior to the diagnosis date were excluded. Among the patients with PDAC, the date of diagnosis was defined as the index date. For each PDAC case, up to 4 controls were selected from a group of patients without PDAC on the index date of the matched cases. Controls could develop PDAC 1 year after the index date. The above study criteria identified a total of 2611 eligible patients with PDAC and 10,085 corresponding matched patients without PDAC during the study period. The study participant identification and NLP process is shown in Figure 1.

### PDAC Symptom Selection

We initially identified 24 PDAC-related symptoms based on literature reviews and clinicians’ input. A survey was conducted among the Consortium for the Study of Pancreatitis, Diabetes, and Pancreatic Cancer working group members [28] to determine the relative importance of the 24 potential symptoms. Based on the ranking of importance, a total of 17 symptoms were finally selected. In this study, we considered abdominal pain and epigastric pain as a combined symptom (abdominal or epigastric pain) and anorexia and early satiety as a combined symptom of (anorexia or early satiety) due to the difficulty of distinguishing them in clinical notes or patient-provider communications. The deep vein thrombosis (DVT) symptom was included in our study because DVT risk is high in patients with pancreatic cancer [29], and the symptom was further delineated into upper and lower DVT.

### PDAC Symptom Keyword Selection

First, we compiled a list of phrases or terms relevant to the 17 symptoms based on previous literature [21-23] or symptom ontologies in the Unified Medical Language System [30]. The list was then reviewed and enriched by the experienced study gastroenterologist and enhanced by manual data annotation processing (refer to “Data Annotation” subsection for details). In addition, we used a word embedding model, Word2vec [31,32], to capture possible relevant phrases and terms, including misspelled terms, for each symptom. The compiled comprehensive phrases and terms for these 17 symptoms are summarized in Table S1 in Multimedia Appendix 1. The PDAC symptoms can be determined by a single phrase or term except for the DVT symptom. The DVT symptom was determined by 3 sets of terms, which included location (eg, leg or arm), feeling or appearance (eg, pain or swollen), and laterality (eg, left or right), rather than a single phrase or term.

### Extraction and Preprocessing of Study Notes

Clinical notes and patient communication messages (telephone or email) within 2 years prior to the index date of PDAC cases and their matched controls (referred to as “notes” hereafter) were extracted from the KPSC EHR system. Notes associated with certain medical encounters (eg, surgery), note types (eg, patient instructions or anesthesia), and department specialties (eg, health education) were excluded from the analysis because symptoms of interest were unlikely to be present in these notes (Table S2 in Multimedia Appendix 1). The extracted notes were then preprocessed through the following steps: (1) lowercase conversion, sentence splitting, and word tokenization [33]; (2) removal of nondigital or nonletter characters except for spaces, periods, commas, question marks, colons, and semicolons; (3) standardization of abbreviated words; and (4) correction of misspelled words based on the Word2vec model supplemented by an internal spelling correction file developed in previous studies [23,25].

### Training, Validation, and Implementation Data Sets

Our study involved 2 phases of training and validation. The first phase used the notes of 100 randomly selected PDAC cases. The second phase used a subset of notes from both PDAC cases and controls. Details of the sample selection for training and validation are summarized in Table S3 in Multimedia Appendix 1. Notes that were not used for training or validation formed the study implementation data set.

### Data Annotation

Notes from both the training and validation data sets were manually reviewed by trained research annotators to indicate the presence of the 17 symptoms based on the established terms and phrases (Table S1 in Multimedia Appendix 1) and inclusion and exclusion criteria (Table S4 in Multimedia Appendix 1). The note annotation process was based on a computer-assisted approach. First, notes from the training and validation data sets were exported into a spreadsheet and the prespecified terms (Table S1 in Multimedia Appendix 1) were highlighted. Second, for each note, the annotators reviewed the notes to label the presence of each of the 17 symptoms. Third, any ambiguous notes were fully discussed during weekly study team meetings until a consensus was reached. Cases that were difficult to determine were reported to the study gastroenterologist for adjudication.

A subset of the training data set in the first phase (n=2795 notes) was double-reviewed (ie, 2 annotators independently reviewed the same set of notes). The results from the 2 annotators were compared and inconsistencies between them were discussed until a consensus was reached. If the annotators did not reach a consensus, the note was reviewed and adjudicated by the study gastroenterologist.

Finally, the adjudicated results were documented as the gold standard for training and validation of the NLP algorithm.

### NLP Algorithm Development

Algorithm development involved 2 phases of training. For each phase, we used the annotated training data set to develop or refine a rule-based computerized algorithm via an iterative process to determine the presence of the 17 symptoms in each note. First, the notes were analyzed based on the phrase or terms and patterns that indicated the presence or absence of each symptom (Table S1 in Multimedia Appendix 1). The algorithm was then processed to search for patterns of inclusion or exclusion to determine the status of each symptom (Table S4 in Multimedia Appendix 1). A list of negated terms (eg, “ruled out” or “negative for”), uncertain or probable terms (eg, “presumably”), definite terms (eg, “positive for”), history terms (eg, “several years ago”), non-patient person terms (eg, referring to a family member), and general description terms (eg, “please return to ED if you have any of the following symptoms”) were compiled from the training data sets. The compiled terms were enriched via the repeated test-revise strategy against the chart review results within each training subset until the algorithm performance reached an acceptable threshold (ie, positive predictive value [PPV]=90%). The discordant cases between the algorithm and manually annotated results for each subset were further reviewed and adjudicated among the annotators and study team until a consensus was reached.

Specifically, each symptom for each note was first determined at the sentence level based on the following criteria:

1. A sentence defaulted as “no” if any exclusion criterion in Table S4 in Multimedia Appendix 1 was met.
2. The symptom was considered absent if the sentence met any of the following situations:
  The sentence did not contain any defined terms listed in Table S1 in Multimedia Appendix 1.
  The negated description was associated with defined terms listed in Table S1 in Multimedia Appendix 1. Examples included “patient denied vomiting/nausea,” “ruled out jaundice,” and “no pruritus.”
  The description of the symptom did not refer to an actual situation. For example, “return if you experience epigastric bloating” and “glipizide side effects including loss of appetite, nausea, vomiting, weight gain.”
  A probable or uncertain description was associated with the symptom. For example, “patient with anxiety and likely depression” and “patient informed that there may be pruritis or pain.”
  The symptoms were associated with a historical term or date relative to the clinical note date. For example, “patient had abdominal pain two years ago” and “patient had jaundice in 2007.”
  The symptom description was related to family history, such as “family history: mother anxiety” and “patient family history: daughter with depression.”
  Someone other than the patient had a symptom. For example, “my husband is in a deep depression” and “daughter-in-law has been stressed, poor appetite and less sleep.”
  The symptom was described as treated by medication during hospitalization.
  The sentence only consisted of a symptom term, so a decision could not be reached on whether this instance was positive for the symptom.
3. A symptom was classified as “yes” for any of the following situations:
  The sentence contained a symptom of interest and the symptom was marked as “yes,” “x,” or “+”. A symptom was classified as “yes” if the response to a symptom question was affirmative or if the symptom was marked on the symptom list.
  The symptom was listed under the diagnosis section (except for DVT), chief complaint section, symptom section, and history of present illness section of the clinical note. For example, “chief complaint: abdominal pain,” “primary encounter diagnosis anxiety disorder,” and “jaundice 782.4.”
  The symptom was described as treated or indicated by medication within nonhospitalization encounters.
  The symptom was documented or reported to be present at the time of visit or messaging. For example, “pt complaint of 55 lb weight loss since March 2009” and “patient here for several weeks of abdominal pain.”
  The sentence contained a definite term associated with a symptom of interest. Examples included “positive for fatigue and weight loss,” “patient reports anorexia,” and “patient presents with anxiety, depression, insomnia.”
4. The sentence-level results were then combined to form note-level results.
  Classification at the note level was defined as “yes” if at least 1 sentence in the note was marked “yes”. Otherwise, it was classified as “no”.

The diagnosis of DVT itself was not considered a DVT symptom. Additionally, the bodily location (ie, source) of pain was considered when determining the presence of any symptom (such as DVT, back pain, or abdominal or epigastric pain). For example, pain *radiating from* the upper or lower extremity was considered a DVT symptom, whereas pain *radiating to* the upper or lower extremity was not. Similarly, pain that *radiated to* the back region was not counted as back pain, and pain that *radiated to* the abdomen or epigastric region was not counted as abdominal or epigastric pain.

### Performance Evaluation

The results of the NLP algorithm against the validation data set were compared to the adjudicated chart review results notes. For each symptom, the numbers of true positive (TP), false positive (FP), true negative (TN), and false negative (FN) cases were used to estimate the sensitivity or recall, specificity, PPV or precision, negative predictive value (NPV), and overall *F*_1_-score, a harmonic balance measurement of PPV and sensitivity. Sensitivity was defined as the number of TPs divided by the total number of symptoms ascertained by the chart reviews (TP+FN). PPV was defined as the number of TPs divided by the total number of symptoms identified by the computerized algorithm (TP+FP). Specificity was defined as the number of TNs divided by the total number of notes without symptoms ascertained by the chart reviews (TN+FP). NPV was defined as the number of TNs divided by the total number of notes identified by the computerized algorithm without symptoms (TN+FN). The *F*_1_-score was calculated as (2 × PPV × sensitivity) / (PPV + sensitivity).

### Interrater Reliability Analysis Among 2 Annotators

The agreement and kappa coefficient against the double-annotated subset were calculated to assess the interrater reliability among the annotators.

### Discrepancy Analysis

For each symptom, discordant results between the NLP algorithm and adjudicated chart review against the validation data set were analyzed. Both FP and FN scenarios were summarized in detail.

### Implementation of the NLP Algorithm

The validated computerized algorithm was implemented via Python programming on a Linux server to process the qualified study notes with the exception of training and validation notes. For each symptom, the process created the results of each note at the sentence level and note level for summary analysis.

## Results

### Statistics of the Study Notes

A total of 408,147 and 709,789 notes were retrieved for 2611 PDAC cases and 10,085 matched controls, respectively. The distribution of the notes and patient demographics are summarized in Table 1. Compared to patients without PDAC, patients with PDAC were older and more likely to be men (PDAC cases: mean 69.2, SD 9.1 years of age and n=1328, 50.9% men; controls: mean 48.6, SD 17.2 years of age and n=4681, 46.4% men). A total of 3,827,166 sentences and 69,455,767 word tokens were derived from notes belonging to patients with PDAC. The corresponding numbers were 5,880,717 sentences and 102,358,031 word token for patients without PDAC. Both the average number of notes per patient and average words per note were higher for patients with PDAC (notes per patient: mean 156.3, SD 138.3; words per note: mean 170.2, SD 319.2) compared to patients without PDAC (notes per patient: mean 70.4, SD 94.1; words per note: mean 144.2, SD 263.6).

**Table 1:** Description of the study population and the associated data sets.

	PDAC^a^ (n=2611)	Non-PDAC (n=10,085)
Age (years), mean (SD)	69.2 (9.1)	48.6 (17.2)
Gender: women, n (%)	1283 (49.1)	5404 (53.6)
Gender: men, n (%)	1328 (50.9)	4681 (46.4)
Total clinical notes, n	408,147	709,789
Total sentences, n	3,827,166	5,880,717
Total word tokens, n	69,455,767	102,358,031
Notes per patient, mean (SD)	156.3 (138.3)	70.4 (94.1)
Sentences per clinical note, mean (SD)	9.4 (15.7)	8.3 (13.9)
Words per clinical note, mean (SD)	170.2 (319.2)	144.2 (263.6)

^a^PDAC: pancreatic ductal adenocarcinoma.

### Interrater Reliability of 2 Annotators

The agreement and kappa coefficient between 2 annotators for a subset of notes (n=2795) is summarized in Table S5 in Multimedia Appendix 1. The agreement ranged from 98.82% (abdominal or epigastric pain) to 99.96% (upper extremity DVT), while the kappa coefficient ranged from 0.6 (insomnia) to 0.91 (abdominal or epigastric pain).

### Validation of the NLP Algorithm

Table 2 summarizes the performance of the computerized NLP algorithm against the adjudicated chart review results of 1000 notes based on the validation data set. In descending order, the precision (PPV) of the algorithms ranged from 98.9% (jaundice) to 84% (lower extremity DVT), recall (sensitivity) ranged from 98.1% (weight loss) to 82.8% (epigastric bloating), specificity ranged from 99.9% (epigastric bloating, jaundice, and pruritus) to 98.9% (depression), NPV ranged from 99.9% (lower extremity DVT) to 98.1% (abdominal or epigastric pain and back pain), and the *F*_1_-score ranged from 0.97 (jaundice) to 0.87 (depression).

**Table 2:** The computerized model’s performance against the adjudicated chart review results in the validation data set (n=1000).

Symptoms		TP^a^ (n)	TN^b^ (n)	FP^c^ (n)	FN^d^ (n)	Sensitivity (%)	PPV^e^ (%)	Specificity (%)	NPV^f^ (%)	*F*_1_-score
**Gastrointestinal symptoms**										
	Abdominal or epigastric pain	156	824	4	16	90.7	97.5	99.5	98.1	0.94
	Anorexia or early satiety	78	909	2	11	87.6	97.5	99.8	98.8	0.92
	Dark urine	51	938	3	8	86.4	94.4	99.7	99.2	0.90
	Epigastric bloating	53	935	1	11	82.8	98.2	99.9	98.8	0.90
	Nausea or vomiting^g^	97	820	3	7	93.3	97	99.6	99.2	0.95
	Pale stool	40	949	5	6	87	88.9	99.5	99.4	0.88
**Systemic symptoms**										
	Back pain	95	882	6	17	84.8	94.1	99.3	98.1	0.89
	Fatigue	105	883	2	10	91.3	98.1	99.8	98.9	0.95
	Jaundice	90	905	1	4	95.7	98.9	99.9	99.6	0.97
	Malaise	52	941	2	5	91.2	96.3	99.8	99.5	0.94
	Pruritus	27	970	1	2	93.1	96.4	99.9	99.8	0.95
	Weight loss	101	886	11	2	98.1	90.2	99.8	99.8	0.94
**Mental symptoms**										
	Anxiety	79	911	3	7	91.9	96.3	99.7	99.2	0.94
	Depression	83	892	10	15	84.7	89.3	98.9	98.3	0.87
	Insomnia	62	925	7	6	91.2	89.9	99.3	99.4	0.91
**Vascular conditions**										
	Lower extremity DVT^h^ symptom	19	977	3	1	95	86.4	99.7	99.9	0.91
	Upper extremity DVT symptom	21	972	4	3	87.5	84	99.6	99.7	0.86

^a^TP: true positive.

^b^TN: true negative.

^c^FP: false positive.

^d^FN: false negative.

^e^PPV: positive predicted value.

^f^NPV: Negative predicted value.

^g^Hospital encounter notes were excluded with the exception of emergency notes.

^h^DVT: deep vein thrombosis.

### Discrepancy Analysis

The discrepancy analysis is summarized in Table S6 in Multimedia Appendix 1. The most common scenarios that resulted in FPs were failure of exclusion of the symptoms described in the patient medical problem list, failure of exclusion of symptoms from instructions, failure of negation, or failure of exclusion of a symptom from past medical history. The most common scenarios for FNs were false negation, missing specific terms or patterns of terms in the search list, false classification of past history symptoms, or false exclusion of symptoms described in relevant medication instructions.

### Implementation of the NLP Algorithm

Table 3 summarizes the symptoms identified by the validated NLP algorithms based on the implementation data set. Of the 393,003 and 708,489 notes belonging to PDAC and non-PDAC patients, respectively, at least 1 symptom was identified in 52,803 (13.44%) and 56,552 (7.98%) notes, respectively. The presence of symptoms ranged (in descending order) from 4.98% (abdominal or epigastric pain) to 0.05% (upper extremity DVT) in patients with PDAC and from 1.75% (back pain) to 0.01% (pale stool) in the patients without PDAC.

**Table 3:** Presence of symptoms identified by the computerized algorithms based on the implementation data set at the clinical note level.

Symptom			Clinical notes from patients with PDAC^a^, n (%) (n=393,003)		Clinical notes from patients without PDAC, n (%) (n=708,489)
Any of 17 symptoms			52,803 (13.44)		56,552 (7.98)
**Gastrointestinal symptoms**					
	Abdominal or epigastric pain	19,582 (4.98)		11,274 (1.59)	
	Anorexia or early satiety	4393 (1.12)		1626 (0.23)	
	Dark urine	1511 (0.38)		121 (0.02)	
	Epigastric bloating	3217 (0.82)		1665 (0.24)	
	Nausea or vomiting	7754 (1.97)		7429 (1.05)	
	Pale stool	875 (0.22)		35 (0.01)	
**Systemic symptoms**					
	Back pain	8407 (2.14)		12,416 (1.75)	
	Fatigue	7170 (1.82)		9621 (1.36)	
	Jaundice	9118 (2.32)		305 (0.04)	
	Malaise	2984 (0.76)		4162 (0.59)	
	Pruritus	1872 (0.48)		622 (0.09)	
	Weight loss	8001 (2.04)		2619 (0.37)	
**Mental symptoms**					
	Anxiety	3924 (1)		10,843 (1.53)	
	Depression	4995 (1.27)		10,810 (1.53)	
	Insomnia	2228 (0.57)		4159 (0.59)	
**Vascular conditions**					
	Lower extremity DVT^b^ symptom	807 (0.21)		1465 (0.21)	
	Upper extremity DVT symptom	215 (0.05)		719 (0.1)	

^a^PDAC: pancreatic ductal adenocarcinoma.

^b^DVT: deep vein thrombosis.

## Discussion

In this study, we developed computerized NLP algorithms to identify 17 symptoms that were documented prior to PDAC diagnosis from clinical notes and patient-provider communication emails. To our knowledge, this is the first study to systematically identify a set of symptoms related to PDAC using NLP. When assessed against the manually annotated results, the algorithm achieved a reasonable performance, with recall (sensitivity) ranging from 82.6% to 98.1% and precision (PPV) ranging from 84% to 98.9%.

Accurate extraction of symptoms embedded in free-text notes posed a significant challenge. First, the symptoms might be described in various portions of the notes. For example, symptoms might be embedded under past medical history, review of systems, the patient’s medical problem list, instructions, sign and symptom warnings, questionnaires, checklists, lab orders and tests, medications, procedures, diagnosis, or chief complaints. Second, health care providers might copy and paste information from previous notes. In addition, we would like to highlight some specific challenges. First, a negated term could sometimes apply to only 1 symptom or to multiple symptoms after negation (eg, no coughing, no chest pain, no abdomen pain; denies nausea or vomiting, diarrhea, constipation, abdominal pain). Second, the defined rules might not address all scenarios. For example, one of our defined rules for abdominal pain required the word “pain” and the body location to be within a 5-word distance. If the words for body location (eg, abdomen) and “pain” were separated by more than 5 words, the sentence was marked “no” for abdominal pain. Third, we found that some symptom terms could have different meanings, which caused FPs. For example, the phrase “lower bp” for back pain could also mean lower blood pressure, and the fatigue term “exhausted” could refer to either physical or mental exhaustion. Fourth, some exclusion criteria, as shown in Table S3 in Multimedia Appendix 1 (eg, exclude localized itching for pruritus), also caused potential misclassification.

The data annotation process was tedious and time-consuming. The following lessons learned could benefit the medical research community. First, set up a training period for chart annotators and study investigators with medical backgrounds to review at least several hundred notes (the same notes for all the annotators). This step would not only allow the chart annotators to be trained for the process but also would identify potential issues that might arise during the formal review process. Second, develop a chart annotation document that would include the detailed inclusion and exclusion criteria to be used for the annotation. The document should define specific types of notes (eg, mental health progress notes) or sections of the notes (eg, “past medical history” or “history of present illness”) to be reviewed or to be skipped. The document should also outline rules to determine the presence or absence of the conditions of interest. For example, if a patient experienced abdominal pain at home but did not experience pain at the time of the visit. Such rules are study-specific, but they need to be considered thoroughly and documented.

Advanced transformer language models, including bidirectional encoder representations from transformers (BERT) [20], clinical BERT [34], BioBERT [35], and BERT for EHRs (BEHRT) [36], have gained popularity in research involving NLP. These NLP language models offer the advantage of contextual understanding through embedding representations, allowing the developed algorithms to capture the meaning and intricate relationships within the text and enhance the accuracy of the analysis. They have been widely used for analyzing information from unstructured notes in the health care domain [18,19,37]. Research in this area in future work is warranted to further boost the performance of PDAC-related symptoms, especially for these lower performances via the rule-based approach.

Our study acknowledged several potential limitations. First, the completeness and accuracy of the extracted symptoms depended on the information documented in the EHR system. Incomplete or inaccurate documentation of symptoms could lead to bias. Second, although our training process was quite comprehensive and included a relatively large number of notes, the rules and lexicons built based on the training data sets were still not highly comprehensive, as summarized in the discrepancy analysis. Therefore, a more extensive sample could be used to enhance the rules and lexicons if applied in other populations in the future, especially for rare symptoms. Third, a few terms or phrases could indicate meanings other than the symptom of interest (eg, “patient has exhausted all conservative measures” or “patient complaint of lower bp than usual”). Additional contexts with these terms would be required to determine the actual meaning. Fourth, for symptoms involving body location, such as abdominal pain and back pain, the allowed distance between the location and the symptom could sometimes lead to the misclassification of TP cases. Lastly, when applied to other health care systems and settings, the developed computerized algorithms might require modifications due to variations in the format and presentation of clinical notes in different health care settings.

In conclusion, the developed computerized algorithm and process could effectively identify relevant symptoms prior to PDAC diagnosis based on unstructured notes in a real-world care setting. This algorithm and process could be used to support the early detection of pancreatic cancer if implemented within a health care system to automatically identify patients with PDAC-related symptoms, especially those with PDAC-specific symptoms.

## Appendix

Multimedia Appendix 1 Supplemental tables.

## Abbreviations

BERT: bidirectional encoder representations from transformers
DVT: deep vein thrombosis
EHR: electronic health record
FN: false negative
FP: false positive
KPSC: Kaiser Permanente Southern California
NLP: natural language processing
NPV: negative predictive value
PDAC: pancreatic ductal adenocarcinoma
PPV: positive predictive value
TN: true negative
TP: true positive
