# Supplementary material for: Identifying Symptoms Prior to Pancreatic Ductal Adenocarcinoma Diagnosis in Real-World Care Settings: Natural Language Processing Approach
Source: JMIR AI. 2024 Jan 15;3:e51240. doi: 10.2196/51240 (PMC11041417; doi:10.2196/51240)
Supplement: Multimedia Appendix 1 [file ai_v3i1e51240_app1.docx]

**Appendixes**

Table S1. Phrases or Terms used for identifying PDAC related symptoms.

| **Symptom** | **Phrases/terms^¶^** |
| --- | --- |
| Abdominal/epigastric pain | - (llq\|rlq\|luq\|ruq\|lq\|uq\|\|left lower quadrant\|right lower quadrant\|left upper quadrant\|right upper quadrant\|lower quadrant\|upper quadrant) (pain\|ache\|cramp\|cramping\|colic) - (abdominal\|abdomen\|\|mid(-\|\s)*epigastric\| epigastric\|gastric\|intestinal\|stomach\|tummy\|gut\|belly\|colicky\|parietal\|pelvic\|visceral) (pain\|ache\|cramp\|cramping\|colic) - (pain\|ache\|cramp\|cramping\|colic) (located )?(in \|at \|under )(the \|my )? (abbdominal\|mid(-\|\s)*epigastric\|epigastric\|gastric\|intestinal\|stomach) - (pain\|ache\|cramp\|cramping\|colic) (located )?(in \|at \|under )?(the \|my )?(llq\|rlq\|luq\|ruq\|lq\|uq\|left lower quadrant\|right lower quadrant\|left upper quadrant\|right upper quadrant\|lower quadrant\|upper quadrant) (abdominal\|abdomen) (region\|area) - pain (loc\|location)\s*:\s*(llq\|rlq\|luq\|ruq\|lq\|uq\|left lower quadrant\|right lower quadrant\|left upper quadrant\|right upper quadrant\|lower quadrant\|upper quadrant\|adominal\|abdomen\|midepigastric\|epigastric\|gastric\|intestinal\|stomach\|tummy\|gut\|belly\|parietal\|pelvic) - epigastric burning - agony in (the \|my )?(abbdominal\|mid(-\|\s)*epigastric\|epigastric\|gastric\|intestinal\|stomach) region - dull (pain\|ache\|cramp\|cramping\|colic) in (the \|my )?tummy - (pain\|ache\|cramp\|cramping\|colic) (in \|at \|under )(the \|my )?rib cage - (burning\|pain\|ache\|cramp\|cramping\|colic) (in \|at \|under )?(the \|my )?abdomen - abdomen:\s*x\s*\d\s*days,\s*(llq\|rlq\|luq\|ruq\|lq\|uq\|left lower quadrant\|right lower quadrant\|left upper quadrant\|right upper quadrant\|lower quadrant\|upper quadrant) (pain\|ache\|cramp\|cramping\|colic) |
| Anorexia/early satiety | - anorexia, anorexic - appetite (loss\|lose\|reduce\|reduction\|decrease\|diminish\|down\|poor) - (lose\|reduce\|reduction\|decrease\|diminish\|lack\|low\|lower\|little\|no\|poor) (appetite\|food intake) - loss of appetite, lack of appetite, decrease in appetite - no longer have appetite - lower energy and appetite - lower energy, appetite - (don’t\|hasn’t) feel (hungry\|like eating) - early satiety - inability to tolerate full meal |
| Dark urine | - (dark\|black\|brown) (color )?urine - (dark\|black\|brown) (color )? but not cloudy urine - smoky urine, coke color urine, tea color urine - dark of urine, dark of (her\|his\|patient) urine - urine( is\| become\| has\| found\| note\| notice\| look\| like\| get\| start\| continue\| turn\| to)*( very\| little\| slight)?(( color)?( ( \|-)*dark\|( \|-)*brown\|( \|-)*black)+\|( ( \|-)*dark\|( \|-)*brown\|( \|-)*black)+ color\| tea color\| ice tea color) |
| Epigastric bloating | - bloat - gas - (abdominal\|abdomen) (distress\|distend\|bloat\|fullness\|pressure\|gas) - abdomen: (distress\|distend\|bloat\|fullness\|pressure\|gas) - (distress\|distend) (abdomen\|stomach) - (mid\s*epigastric\|epigastric) (distress\|distend\|bloat\|fullness\|pressure\|gas\|swell) - (pressure\|fullness\|distress\|distention) (located )?(in \|under \|at )?(the \|my )?(llq\|rlq\|luq\|ruq\|lq\|uq\|left lower quadrant\|right lower quadrant\|left upper quadrant\|right upper quadrant\|lower quadrant\|upper quadrant\|upper left part of\|lower left part of\|upper right part of\|lower right part of\|upper\|lower) abdomen - dilatation of stomach - inflamado - inflammacion - flatulence |
| Nausea/vomiting | - n/v, n-v, n&v, n/v/d, n-v-d, n&v&d, nvd - nausea - vomit - throwing up - emesis - barf - puke, - upchuck |
| Pale stool | - (pale\|acholic\| clay( \|-)+color\| color light\|gray\|grey\|white\|light color\|light) (feces\|stool\|bowel movement) - (feces\|stool\|bowel movement) (pale\|acholic\| clay( \|-)+color\| color light\|gray\|grey\|white\|light color\|light) - light (feces\|stool\|bowel movement) color - light (brown\|beige)+ color (feces\|stool\|bowel movement) - (feces\|stool\|bowel movement) (color )?(is\|turn\|become\|change) (to )?(pale\|(light )?clay\|(light )?gray\|(light )?grey\|white) |
| Back pain | - back\s*(pain\|ache\|aching\|sore\|soreness\|strain) - lbp, ubp, lower bp, upper bp, middle bp, mid\W*bp - (low back\|lower back\|upper back\|middle back\|\|mid(-\|\s)*back\|the back\|region of back\|his back\|her back\|my back\|entire back\|lumbar spine\|lumbar) (pain\|ache\|aching\|sore\|soreness\|muscle spasm\|muscle cramp) - (pain\|ache\|aching\|sore\|soreness\|muscle spasm\|muscle cramp) (located )?(in \|at \|under )(the \|my )? (lowe?r? back\|upper back\|middle back\|mid(-\|\s)*back\|the back\|region of back\|his back\|her back\|my back\|entire back\|lumbar region of back\| s\|back left side\|back right side\|)?(left \|lt? \|right \|rt? \|middle \|mid(-\|\s)* )?(lower\|upper) back\|lb\|ub) - (pain\|ache\|aching\|sore\|soreness) between shoulder blades - back become (very \|excruciate \|extremely \|so )?painful - myofascial pain syndrome of (lb\|ub\|lower back\|upper back\|mid(-\|\s)*back) - spasm of muscle of (lb\|ub\|lower back\|upper back\|mid(-\|\s)*back) - lumbo( \|-)*sacral (pain\|strain) - low back [1-10]/10 pain |
| Fatigue | - fatigue - tired, tired out - lethargy - weary, worn out - exhausted - asthenia - asthenic - knackered - tatt - enervated - listless - somnolence - (lack of\|loss of\|lack\|loss\|lose\|no\|low\|lower\|very little\|not much) energy - energy level:(very \|much \|pretty \|quite )?low - don’t have energy - general weakness, weakness in general, \|weakness generally - decrease (in )?energy - (feel\|get) run down |
| Jaundice | - jaundice - icteric - icterus - yellow complexion - yellowish - yellow (skin\|sclera\|eye\|eyeball\|eyelid) - (skin\|sclera\|eye\|eyeball\|eyelid) (is\|become\|start\|look\|increase\|appear\|get\|turn\|develop\|has) yellow - (skin\|sclera\|eye\|eyeball\|eyelid) yellow |
| Malaise | - Malaise - Sick feeling - feel (sick\|ill\|un(-\|\s)*well\|not well\|worse\|a bit (of )?worse\|a little bit (of )?worse\|bad) - not feel (well\|good) - (isn’t\|don’t\|hasn’t) feel (well\|good) - (isn’t\|hasn’t) been (feeling\|felt) (well\|good) |
| Pruritus | - pruritus, pruritic - itch, itchy, itchiness |
| Weight loss | - (weight\|\d*lbs?\|\d*pounds?\|\d*kgs?) (lose\|reduce\|decrease\|go down\|cut down\|lower down) - (lose\|reduce\|decrease) ((in )?(body )?weight\|\d*\s*lbs?\|\d*\s*pounds?\|\d*\s*kgs?) - (lose\|reduce\|decrease) (about )?\d+\s*-\s*(\d*\s*lbs?\|\d*\s*pounds?\|\d*\s*kgs?) - (go\|cut\|lower) ((body )?weight\|\d*\s*lbs?\|\d*\s*pounds?\|\d*\s*kgs?) down - (go\|cut\|lower) (about )?\d+\s*-\s*(\d*\s*lbs?\|\d*\s*pounds?\|\d*\s*kgs?) down - weight is down - weight has (be \|being \|been )?down - down \d+\s*(lbs?\|pounds?\|kgs?) - weight is (\d+\s*lbs?\|\d+\s*pounds?\|\d+\s*kgs?) less than - weight change is\s*-\s*\d\d+(\.\d+)?\s*(lb\|pound\|\#\|kg)s? - lose (quite \|pretty )?(a bit of \|a bit \|a little of \|a little \|some )?weight - waste away |
| Anxiety | - anxiety - panic attack - panic disorder - panic feeling - feel (scared and \|fear and )?panic - agora\W*phobia - phobia,\s*social - social phobia |
| Depression | - depression - depressive disorder - depressive episode - depressive symptom - (quite\|very\|too\|pretty\|so\|completely\|fully\|entirely\|totally\|wholly\|absolutely\|utterly\|thoroughly\|somewhat\|more\|much\|so much\|really\|less) (depressed\|depressful) - (feel\|seem) (depressed\|depressful) - (he\|she\|patient) (is \|has been) (so )?(depressed\|depressful) - i am (so )?(depressed\|depressful) - mood:\s*(depressed\|depressful) - mood (depressed\|depressful) - (depressed\|depressful) mood - dysthymia - melancholia |
| Insomnia | - insomnia - sleeplessness - sleepless night - sleep initiation and maintenance disorder - (difficulty\|trouble\|problem) (with )?sleep - difficult (falling \|getting )?to sleep - difficulty (get\|fall\|stay) asleep - lying awake for a long time at night - trouble going back to sleep - not (be \|been \|being ) ?sleep - (can’t\|loss of\|lose\|trouble\|poor\|not good\|lack of) (with \|w )?sleep - (inability\|inable\|unable\|not able) to (get )?(good )?sleep - (isn’t\|don’t\|hasn’t\|can’t) get enough sleep - (isn’t\|don’t\|hasn’t\|can’t) help (her \|his \|patient )?sleep - ambien for sleep - sleep (with\|on) ambien |
| Lower extremity DVT symptom | - Location: leg, thigh, lower limb, calf, lower extremity, le, lle, rle - Feeling/appearance: pain, ache, warm, swell, edema, hot - Laterality: right, left, lt, rt, r, l, unilateral, one |
| Upper extremity DVT symptom | - Location: arm, forearm, tricep, bicep, supinator, forelimb, upper limb, upper extremity, ue, lue, rue - Feeling/appearance: pain, ache, warm, swell, edema, hot - Laterality: right, left, lt, rt, r, l, unilateral, one |

¶ Also search lemma variants (adjective, adverb, plural form and verb tenses) and abbreviations of these words, where applicable.

‘|’ stands for any word/phrase in the parentheses

‘?’ stands for the prior character/phrase in the parentheses can be zero or one

‘*’ stands for the prior character/phrase in the parentheses can be zero or any

‘+’ stands for the prior character/phrase in the parentheses can be one or more

‘\s’ stands for empty space

‘\W’ stands for non-letter/digital character

Table S2. Exclusion of encounters, specialty, clinical note types used for identifying PDAC related symptoms.

| **Encounter** | Anesthesia, Future or stand, Surgery |
| --- | --- |
| **Specialty** | Anesthesiology, Audiology, Bariatric medicine, Blood donor service, Cosmetics, Employee health, Health education, Mohs surgery services, Neurosurgery, Ophthalmology, Optometry, Orthopaedics surgery of the spine, Otolaryngology, Pharmacy, Spine center, Surgery ambulatory, Surgery cardiovascular, Surgery general, Surgery maxillofacial, Surgery vascular, Transplant heart, Transplant liver, Transplant lung, Wound care |
| **Note type** | Anesthesia preprocedure evaluation, Anesthesia postprocedure evaluation, Anesthesia preop, Anesthesia procedure notes, Anesthesia info, Anesthesia postop, Anesthesia procedure, Anesthesia followup, Anesthesia pain management, OR nursing, OR preop, OR surgeon, OR postop, OR anesthesia, Letter, L&D delivery note, MR avs snapshot, z-bioethics progress note, Patient instruction, Discharge instruction |

Table S3. The details of training dataset and validation dataset.

| Phase 1 | - Randomly selected 100 PDAC cases with a total of 13,844 study interested notes - Divided these notes into 4 batches, each contains 25 PDAC cases - The notes of first three subsets used for training - The notes of the last for validation |
| --- | --- |
| Phase 2 | - Selected 1,000 notes from PDAC cases and controls for training   - For each symptom, 50 notes (25 from cases and 25 from controls) were randomly selected from notes with pre-identified terms in eTable 1.   - Randomly selected 150 notes from those without any study specific terms (75 from cases and 75 from controls) - Continued to improve algorithms for pruritus, lower extremity DVT and upper extremity DVT by using 600 randomly selected NLP identified symptom notes (200 for each symptom, 100 from case and 100 from control) - Selected 1,000 notes from PDAC cases and controls for validation   - For each symptom, 50 notes (25 from cases and 25 from controls) were randomly selected from notes with pre-identified terms in eTable 1.   - Randomly selected 150 notes from those without any study specific terms (75 from cases and 75 from controls) |

Table S4. Inclusion and exclusion criteria for PDAC related symptoms

| **Criteria** | | **Condition** |
| --- | --- | --- |
| Inclusion | | - At least one appearance of the keywords/phrases in the two years prior to the index date - Symptom indicated by a medication is included only when the stated purpose is for treating the symptom |
| Exclusion | All symptoms | - Symptom appeared in patient active problem list, except hospital active problem list - Symptom appeared in past medical history - Symptom appeared in instruction (e.g., patient instruction, medication instruction, discharge instruction) - Symptom indicated by medication usage but prescribed as prn or as needed - Symptom indicated by side effects |
|  | Weight loss | - Intentional (in known), such as, regular exercises, on diet, etc. - In a weight loss program, any time during the two years prior to the index date |
|  | DVT symptoms | - Bilateral - Symptom appeared in physical therapy or occupational therapy notes - Trauma, surgery, injury, accident, wound, fall, hits, slam, bite, skin tear |
|  | Pruritus | - Localized - Bite, sting, rash, hives, mole, jock itch, swollen wound, injection, bump, yeast, blister, shot, spot, lesion, red patch, incision |
|  | Nausea/Vomiting | - Hospital notes except for emergency room (ER) notes |

Table S5. Agreement and kappa coefficient of a training subset notes (n=2,795) among 25 PDAC patients between two research annotators.

| **Symptom** | **Agreement** | **Kappa coefficient and 95% confidential interval** |
| --- | --- | --- |
| Abdominal/epigastric pain | 98.82% | 0.91 [0.88, 0.94] |
| Jaundice | 99.07% | 0.91 [0.87, 0.94] |
| Anorexia/early satiety | 99.50% | 0.80 [0.70, 0.90] |
| Nausea/vomiting | 99.18% | 0.86 [0.81, 0.92] |
| Anxiety | 99.68% | 0.85 [0.75, 0.95] |
| Weight loss | 99.46% | 0.84 [0.76, 0.92] |
| Pruritus | 99.71% | 0.83 [0.72, 0.95] |
| Back pain | 98.93% | 0.82 [0.76, 0.88] |
| Malaise | 99.71% | 0.81 [0.68, 0.94] |
| Fatigue | 99.43% | 0.80 [0.70, 0.89] |
| Upper extremity DVT symptom | 99.96% | 0.80 [0.42, 1.00] |
| Epigastric bloating | 99.46% | 0.78 [0.67, 0.89] |
| Pale stool | 99.75% | 0.77 [0.61, 0.94] |
| Lower extremity DVT symptom | 99.46% | 0.70 [0.56, 0.85] |
| Depression | 99.46% | 0.66 [0.50, 0.82] |
| Dark urine | 99.21% | 0.65 [0.51, 0.79] |
| Insomnia | 99.57% | 0.60 [0.39, 0.81] |

Table S6. Discrepancy analysis of false positives and negative positives against the validation dataset using the adjudicated chart review results as the reference standard.

| **Symptom** | **Number of cases** | **Type of Misclassification** |
| --- | --- | --- |
| **False positive** | | |
| Abdominal/epigastric pain | 1 | Symptom occurred during procedure/exam/test |
|  | 1 | Conflict positive and negated instances appeared in same note |
|  | 1 | False treated symptom in a list of symptoms without highlight as positive |
|  | 1 | Pain described for other location rather than abdominal/epigastric |
| Anorexia/early satiety | 2 | Failure of negation |
| Dark urine | 2 | Failure of exclusion due to combination of dark with other color |
|  | 1 | Failure of exclusion for history symptom |
| Epigastric bloating | 1 | Failure of exclusion gas described other location |
| Nausea/vomiting | 2 | Failure of exclusion for symptom in instruction |
|  | 1 | Failure of exclusion for history symptom |
| Pale stool | 2 | Failure of exclusion for lab test with term |
|  | 2 | Failure of exclusion for stool with mixed colors |
|  | 1 | Failure of exclusion for medication instruction |
| Back pain | 2 | Failure of exclusion for symptom in problem list |
|  | 3 | Failure of exclusion for history symptom |
|  | 1 | Abbreviated lower bp standard for lower blood pressure rather than lower back pain |
| Fatigue | 1 | Failure of negation |
|  | 1 | Term "exhausted" had different meaning rather than fatigue |
| Jaundice | 1 | Failure of negation |
| Malaise | 2 | Failure of exclusion terms indicated something else rather than malaise |
| Pruritus | 1 | Failure of exclusion for localized itching |
| Weight loss | 4 | Failure of exclusion for intentional weight loss |
|  | 2 | Failure of exclusion for symptom in problem list |
|  | 2 | Failure of negation |
|  | 1 | Failure of exclusion due to conflict positive and negative terms |
|  | 1 | Failure of exclusion for term in check list rather than actual symptom |
|  | 1 | Failure of exclusion for weight loss calculated by vital sign measurement |
| Anxiety | 1 | Failure of exclusion for history symptom |
|  | 2 | Failure of excluded symptom in problem list |
| Depression | 5 | Failure of history classification |
|  | 3 | Failure of exclusion for symptom in problem list |
|  | 1 | Failure of exclusion for term in allergy section |
|  | 1 | Failure of exclusion term in a survey questionnaire |
| Insomnia | 5 | Failure of exclusion for term "cant sleep" due to specific reason rather than insomnia |
|  | 1 | Failure of exclusion for term "poor sleep", which don’t mean insomnia |
|  | 1 | Failure of exclusion for term in check list rather than actual symptom |
| Lower extremity – DVT symptom | 1 | Failure of exclusion for symptom due to surgery |
|  | 1 | Failure of exclusion for symptom due to injury |
|  | 1 | Failure of exclusion for symptom mixed with other body location |
| Upper extremity – DVT symptom | 1 | Failure of exclusion for symptom due to surgery |
|  | 1 | Failure of exclusion for symptom due to picc placement |
|  | 1 | Failure of exclusion for symptom due to injury |
|  | 1 | Failure of negation |
| **False negative** | | |
| Abdominal/epigastric pain | 7 | Term not in existed search terms |
|  | 4 | False negation |
|  | 3 | Term appeared in excluded discharge instruction section |
|  | 1 | False classification as history symptom |
|  | 1 | Term not in existed search terms |
| Anorexia/early satiety | 4 | False negation |
|  | 4 | Term not in existed search terms |
|  | 3 | False classification as history symptom |
| Dark urine | 3 | Terms not in existed search terms |
|  | 2 | False classification as history symptom |
|  | 1 | False negation |
|  | 1 | False exclusion due to sentence only contained keyword, unable to determine |
|  | 1 | False exclusion due to combination of dark with other color |
| Epigastric bloating | 5 | False negation |
|  | 4 | Term not in existed search terms |
|  | 1 | False exclusion due to sentence combined with medication instruction |
|  | 1 | False exclusion due to sentence only contained term, unable to determine |
| Nausea/vomiting | 7 | False negation |
|  | 1 | False classification as uncertain symptom |
| Pale stool | 2 | False negation |
|  | 1 | False history classification |
|  | 2 | Term not in existed search term |
|  | 1 | False exclusion by stool with mixed colors |
| Back pain | 8 | False exclusion by pain mixed with other location |
|  | 3 | False exclusion by sentence contained only term, unable to determine |
|  | 3 | False negation |
|  | 1 | False history classification |
|  | 1 | False exclusion due to term in medication instruction |
|  | 1 | Term not in existed search term |
| Fatigue | 4 | False negation |
|  | 2 | Term not in search terms |
|  | 1 | False probable classification |
|  | 1 | False non-patient classification |
|  | 1 | term "exhausted" with different meaning rather than fatigue |
|  | 1 | False exclusion by sentence contained only term, unable to determine |
| Jaundice | 2 | Term pattern not in existed search term list |
|  | 1 | False negation |
|  | 1 | False exclusion due to “if condition” general statement |
| Malaise | 2 | False negation |
|  | 2 | False exclusion due to sentence combined with medication instruction |
|  | 1 | False exclusion by sentence contained only term, unable to determine |
| Pruritus | 2 | False exclusion for localized itching |
| Weight loss | 1 | False negation |
|  | 1 | False exclusion by sentence contained only term, unable to determine |
| Anxiety | 2 | False negation |
|  | 1 | False classification as history symptom |
|  | 1 | False exclusion as medical side effect |
|  | 1 | False exclusion as medical instruction |
|  | 2 | symptom in general goal statement |
| Depression | 7 | False negation |
|  | 4 | False history classification |
|  | 3 | Term not in search term list |
|  | 1 | False exclusion for term in medication instruction |
| Insomnia | 1 | False exclusion due to sentence combined with medication instruction |
|  | 1 | False negation |
|  | 4 | False exclusion because of term in medication instruction |
| Lower extremity – DVT symptom | 1 | Term pattern not in existed in term search list |
| Upper extremity – DVT symptom | 2 | Term pattern not in existed term search list |
|  | 1 | False exclusion due to surgery |
